# New technologies for safe practice and training during pregnancy: a gynecologist and forensic pathologist perspective narrative review

**DOI:** 10.3389/fsurg.2025.1513832

**Published:** 2025-01-17

**Authors:** Emanuele Capasso, Mariagrazia Marisei, Carmen Imma Aquino, Pierpaolo Di Lorenzo, Claudia Casella

**Affiliations:** ^1^ Department of Advanced Biomedical Sciences, School of Medicine and Surgery, University of Naples Federico II, Napoli, Italy; ^2^ Department of Translational Medicine, Università del Piemonte Orientale, Gynecology and Obstetrics, AOU “Maggiore della Carità”, Novara, Italy

**Keywords:** pregnancy, residency, obstetrics and gynecology training, forensic pathology training, new technologies, metaverse

## Abstract

This paper investigates the intersection of gender health equity within two distinct medical residency programs: obstetrics &gynecology and forensic pathology. It specifically addresses the unique challenges that young female residents encounter during pregnancy, emphasizing the Italian regulatory frameworks and recommendations pertinent to both fields. The online search for this narrative review was based on keywords such as: “medicine” AND “education” AND “pregnancy” on the main online scientific database along with “metaverse” AND “education” AND “pregnancy”. The analysis reveals the heightened risks faced by female medical doctors in surgical and forensic environments, underlining the need for targeted support. Additionally, this study explores the perceived potential of augmented reality (AR) and the metaverse as innovative solutions to mitigate biological risks, thereby enabling pregnant surgeons to maintain effective practice during this critical period. The findings obtained from a literature review aim to contribute to ongoing discussions about improving gender equity in medical training and practice, regarding the surgical and forensic field where the temporary stopping period may result in a different surgical learning curve compared to that of male colleagues.

## Introduction

Gender health equity is a critical issue in healthcare and education, particularly in surgical fields where disparities persist (1–4). The culture of delay of pregnancy among female practitioners begins in the phases of pre-graduation training, when it would be the desirable biological age (5) and persists for the training phase based on population studies conducted (6, 7). In general, it is a real slippage of personal decisions, which tend to be taken late compared with non-medical women in view of a conflict with career goals (8). Many physicians both in their residency programs or immediately after, decide to postpone childbirth, often recurring to oocyte cryopreservation (9). Therefore, social freezing, a process where individuals choose to freeze their eggs or embryos for future use, has become increasingly relevant also for women pursuing medical careers, as it allows female medical professionals to extend their fertility. In general, the set of challenges for both surgical and forensic pathology residents are largely overlapping and can be summarized in exposure to chemical, physical and biological hazards, physical strain and psychological stress. The two residency programs (obstetrics &gynecology and forensic pathology) have been chosen for implications in legislative terms and for their similarity in requiring that part of the training is specifically dedicated to surgical procedures, yet one performed in surgical room and the other in autopsy room. The results of a 2021 Italian ministerial survey on the opinion of medical and surgical residents enrolled from the second year of the course have been recently published. The survey was conducted using CAWI (Computer-Assisted Web Interviewing). Specific questions were asked with the objective of investigating maternity/parenting protection. To the question: “Are you aware of any situations in your school where incidents of non-recognition of compulsory or optional maternity/paternity leave have occurred?” the percentages of positive responses were 0.3% for Obstetrics and 3.2% for Forensic Medicine. Incidents of non-recognition of breastfeeding or other daily rest allowances were reported from 2.2% of Obstetrics and Gynecology residents and 1.9% of Forensic Medicine residents who reported incidents of non-recognition of breastfeeding or other daily rest allowances (10). Working with patients or handling dead bodies (as for Forensic Pathologist) can expose residents to infectious diseases, including bloodborne pathogens (e.g., HIV, hepatitis B and C). However, forensic pathologists have additional issues related to working with cadavers and biological materials. In addition to this the use of formaldehyde and other chemicals in autopsy procedures may pose specific risks. Both disciplines can lead to physical strain, particularly during surgical sessions or autopsies, because of heavy lifting or long hours at work.

## Materials and methods

**Figure 1:**
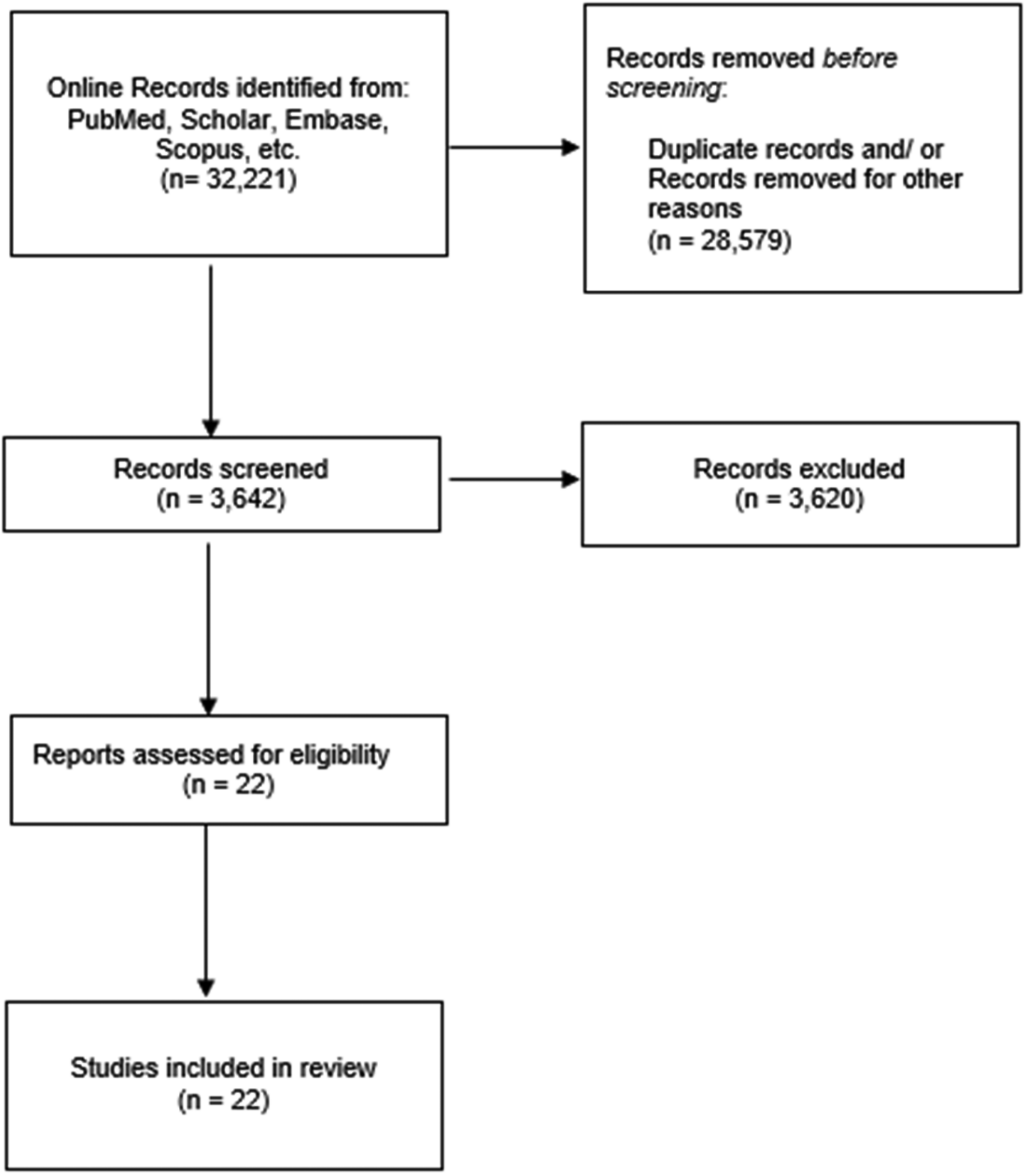
Flowchart. The diagram details our search and selection process applied during the overview. Figure related to “pregnancy” AND “education” AND “medicine”. No results reported about “metaverse” AND “education” AND “pregnancy”.

A preliminary global analysis about the technologies for safe practice and training during pregnancy was carried out based on research gathered by Pubmed, Scholar, Embase, Scopus, etc. The identification of 32,221 articles was based on the keywords “medicine” AND “education” AND/OR “pregnancy” without time limit. 3,642 Articles in the English language were filtered according to the evidence of the scientific research (based on the strength of the evidence, sample size, quality of the studies, etc.). 3,620 Duplicates, research in other languages than English, data not specific or not relevant for the topic (i.e., about gynecological diseases or other forensic aspects) were excluded (Flowchart, Figure 1). Our narrative review is written following PRIOR Guidelines (11) (Supplementary Material).

## Results

The 22 articles in our review are only related to the online search about “pregnancy” AND “education” AND “medicine”. The literature highlighted no results about “metaverse” AND “education” AND “pregnancy” (Table 1).

**Table 1:** Literature review results using the pubMed database.

Keywords	Search mode	Results
Metaverse AND education AND medicine	Metaverse (text word) AND education (Text word) AND medicine (Text word)	3,642
Metaverse AND education AND pregnancy	Metaverse (text word) AND education (Text word) AND pregnancy (Text word)	0

## Discussion

The targeted literature review conducted shows that a real cultural shift is required to reconcile motherhood and a surgical career. with still little attention paid to the potential of new technological tools when specifically applied to the training of female residents in the areas of training with the highest risk for pregnancy. Results from a survey conducted among young female physicians to assess fertility, childbearing history, reflections regarding decision-making, and perceptions of workplace support, showed that 28.6% would have attempted conception earlier, 17.1% would have gone into a different specialty, and 7.0% would have used cryopreservation to extend fertility (12). Compared with male surgeons, female surgeons were more likely to delay having children because of surgical training and were more likely to use assisted reproductive technology. Compared with female non-surgeon partners, female surgeons were more likely to have major pregnancy complications, especially those operating 12 or more hours per week (13). Discriminations regarding career goals has been explored in a survey by Giantini et al. that demonstrated that a notably higher number of women expressed worries regarding the time available to date or marry (*P* = 0.042), the time they could spend with family (*P* = 0.015), the ability to find time to have a child during residency (*P* < 0.0001), the challenges of taking maternity or paternity leave while in residency (*P* < 0.0001), and concerns about being too old to have a child after completing residency (*P* < 0.0001) (14). Pregnant medical residents often face significant stress due to the physical demands of their training and a lack of support from colleagues and their department (15). It's since 1986 that it has been observed that if proper arrangements were made, pregnancy would not be a major problem for either the training program or the pregnant residents (16). Nevertheless, according to Rangel (17) a total of 135 interviewed (39.0%) strongly considered leaving surgical residency, and 102 (29.5%) would discourage female medical students from a surgical career, specifically because of the difficulties of balancing pregnancy and motherhood with training. Female surgeons face specific reproductive hazards that can impact their fertility and pregnancy outcomes pertaining both to intrinsic factors, such as delayed childbearing that involves age related fertility issues or extrinsic factors such as mechanical or biological hazards (18, 19). Forensic pathology is a field for which no data is available, although hazards and stress perceived might be like that of training surgeons. Surgical specialties have specific challenges related to the rigor of training, length and cultural factors related to a masculine interpretation of surgical specialties (19, 20); Forensic Pathology seems to be not seen as a male specialty. Flexible career pathways and work patterns are required to overcome the cultural barrier in surgical specialties (20). In Italy, there are specific regulations designed to protect pregnant workers, including those in forensic pathology. Regulations surrounding pregnancy and maternity leave for female surgeons and healthcare professionals are structured to ensure both health equity and workplace safety. There is a complete prohibition for pregnant workers to be exposed to some physical, chemical and biological agents. Italian regulation applicable to pregnant female healthcare workers is analyzed in detail. Law n 151/2001 (21), Article 7, prohibits assigning female workers to tasks involving the transport and lifting of weights, as well as to dangerous, strenuous, and unhealthy jobs. Among these hazardous tasks are those that pose risks of exposure to agents and working conditions listed in Annex B. During the period of prohibition, the worker will be assigned to other duties. Biological agents classified as risk groups 2, 3, and 4 according to Article 268 and Annex XLVI of Legislative Decree No. 81 of April 9, 2008 (22), are also included (Table 2).

**Table 2:** Classification of biological agents based on their infection risk.

Risk group	Description
1	Biological agents that present a low probability of causing disease in humans.
2	Biological agents that can cause diseases in humans and pose a risk to workers; unlikely to spread in the community; effective preventive or therapeutic measures are usually available.
3	Biological agents that can cause serious diseases in humans and pose a significant risk to workers; may spread in the community, but effective preventive or therapeutic measures are typically available.
4	Biological agents that can cause severe diseases in humans, pose a serious risk to workers, and have a high potential for community spread; effective preventive or therapeutic measures are generally not available.

Apart from these absolute prohibitions there are many protection measures that tend to support maternity. For example, under the Italian budget law effective from January 1, 2019 (23), pregnant women can choose to work until childbirth if certified healthy by their doctor. They are entitled to five months of compulsory maternity leave following the birth of their child. This is a significant change from previous regulations, which divided maternity leave into two segments: two months before and three months after childbirth. Compulsory maternity leave is compensated at 80% of the salary used to calculate social security contributions. Pregnant employees cannot be dismissed from their jobs from the start of pregnancy until the child is one year old, with very few exceptions. Employers are required to adjust tasks for pregnant employees in physically demanding roles to ensure their safety and that of the unborn child. The privileged care attributed to pregnant women is suggested by the peculiar attention given by several directives of the Council of Europe that also supports maternity leave and breastfeeding. No one can force a pregnant woman to work in risky conditions or to work at night (24–27). Differently from European context, in US there is not a specific ban for pregnant women to perform surgery and continue their trainee program. In fact, operating during pregnancy may be safe if the right conditions are met (28). In the majority of OECD nations (Organization for Economic Co-operation and Development), paid parental leave with employment protection is a key component of family policy. By allowing both parents to take time off from paid employment to care for a very young child, paid parental leave primarily seeks to support parents and kids. An overview of parental leave policies and their implementation in OECD nations may be found in the OECD Family Database. All but one OECD nation provides paid maternity and paternity leave during childbirth at the national level as of April 2022; however, the length, cost, and adoption of these laws vary greatly throughout nations. Paid maternity leave lasts 18.5 weeks on average throughout the OECD, but it can vary from 43 weeks in Greece to none at all in the US (29). In the United States there has been the intervention of the Accreditation Council for Graduate Medical Education (ACGME) that has obtained the requirement of lactation facilities and the mandatory leaving for at least 6 weeks for all residency boards. In August 2020, the American Community Survey (ACS) and the ACGME Residency Review Committee released a statement outlining the following measures to protect pregnant residents: the residency program should support trainees' medical needs; it should design a flexible and equitable schedule that allows trainees to take time off while taking into account the needs of other health care professionals; and it should offer at least six weeks of paid parental leave for either or both parents (30). A schematic representation of the comparison between US and EU is provided in Table 3.

**Table 3:** Comparison between US and EU on the protection of pregnant workers.

Countries analyzed	US	EU
Regulations adopted	• Family and Medical Leave Act (1993) (31). • Pregnant workers Fairness Act (2023) (32).	Council Directive 92/85/EEC of 19 October 1992 on the introduction of measures to encourage improvements in the safety and health at work of pregnant workers and workers who have recently given birth or are breastfeeding (33).
Ban for pregnant workers	No specific ban for risky activities.	specific ban for risky activities.
Adjustment policy	Reasonable accommodation required.	Adjustment of the workplace often regulated by law in several Member States.

Up to 12 weeks of unpaid, job-protected leave are granted to qualified employees annually under the Family and Medical Leave Act (FMLA) (31). It also mandates that their group health benefits be maintained while they are on leave. Companies with 50 or more employees, as well as all public and private primary and secondary institutions, are covered by the FMLA. For any of the following reasons, these employers are required to offer up to 12 weeks of unpaid leave annually to an eligible employee: for the birth and upbringing of a worker's newborn; for a kid in foster care or adoption to be placed with the staff; to take care of a parent, spouse, kid, or other member of one's immediate family who has a serious illness; or to take medical leave when an employee is unable to work due to a serious illness. In addition to this, more recently, the Pregnant workers Fairness Act (PWFA) for eligible candidates or workers with known restrictions, the PWFA offers appropriate accommodations. Physical or mental conditions associated with, impacted by, or resulting from pregnancy, childbirth, or related medical conditions are considered “limitations” under the PWFA (32). “Reasonable accommodations” are changes that could be provided in the work environment or the way things are usually done at work. Nevertheless, if making a reasonable accommodation would put an unreasonable burden on the employer, the PWFA does not require the employer to do so (“undue hardship”). Differently from the US, in Europe the discipline is more stringent (33), since risky activities are outlined by law and divided into generic and specific hazards. Generic hazards include nocturnal work, hazards resulting from inappropriate rest/nutrition/hygene, standing activities, sitting activities, occupational stress, etc. whereas specific hazards include risk deriving from all chemical, biological and physical agents. Employers must take action to protect female employees when hazards are discovered, for as by giving them leave or relocating them to a different role. Workers who are pregnant are exempt from working night shifts as long as they can provide a medical certificate. During working hours, pregnant employees are permitted to have prenatal medical exams without losing their wages. A minimum of 14 consecutive weeks of maternity leave, including at least two weeks of mandatory maternity leave, are required under the regulation before and/or after delivery. There are safeguards against every risk mentioned by the European directive and employers are responsible for choosing the measures that best suit their needs while adhering to local, state, and federal laws.

Despite this important set of institutional protection for pregnant workers, residents need also to potentiate their practice during gestational period and continue their training. It would be possible with specific tools, as metaverse. From the research conducted, there are 0 articles with metaverse related to healthcare education in pregnancy as shown in Table 1. On the other hand, thousands of articles in the last few years analyzed the use of metaverse for training purposes in medicine. The results demonstrate that the analyzed regulations are particularly restrictive, effectively prohibiting pregnant women from accessing environments that pose health risks. Scientific societies and the broader scientific community continue to discuss achieving equitable training standards for pregnant women (34), given the prevailing culture that views time spent away from practice as time lost (35). While there are objective limitations in identifying options to adapt to careers, there is also a pressing need to identify informed policies to guide workplace adaptations for physicians who are pregnant (36). New technologies possess substantial potential to transform healthcare significantly. By integrating with the Internet of Medical Devices, quantum computing, and robotics, the metaverse is positioned to redefine healthcare systems (37). The metaverse offers limitless possibilities and is expected to influence future advancements in medical science and technology (38). Telemedicine can greatly diminish barriers for patients seeking medical treatment, enabling healthcare providers to overcome geographical constraints and optimize the use of medical resources. Furthermore, sharing patient data can facilitate advancements in clinical research (38). Nevertheless, until this moment, metaverse and augmented reality have been analyzed for their specific potential for medical (39) and surgical training without a gender-based perspective. Pregnant surgeons could benefit from AR and metaverse by participating in surgeries through remote guidance without compromising safety. Surgeons can practice skills and collaborate remotely without physical constraints. This immersive platform would allow pregnant surgeons to engage in training and mentoring without direct exposure to surgical risks. Pregnant surgeons can engage in surgical procedures through AR, minimizing physical strain and exposure to hazardous conditions. Telemedicine also appears as a great tool to continue receiving patients, letting pregnant women continue their activity even in case of presence of a biohazard that discourages direct contact with patients. These technologies could reduce exposure to harmful agents while maintaining surgical proficiency, although no data is available in literature.

## Conclusions

The intersection of gender health equity in surgical practices and forensic pathology reveals significant gaps in addressing the unique challenges faced by young female surgeons during pregnancy. By comparing regulations across both fields, we identify critical areas for improvement. Furthermore, technological advancements such as AR and the metaverse present promising solutions that allow pregnant surgeons to continue their practice, while minimizing biological risks. Future efforts should focus on developing comprehensive guidelines that prioritize gender equity and leverage technology to create safer working environments for all healthcare professionals. Most of the papers concentrate on the repercussions of metaverse on training without a specific gender-based approach. Gynecology and forensic pathology are united by the important role attributed to anatomy, that requires constant practice in surgical and sector room and the biological risk that occurs during pregnancy. For both residency programs, as for all pregnant healthcare professionals, exposure to biological risk is forbidden during pregnancy. Even if metaverse has been largely analyzed, especially in the last few years regarding its applications in education, there is still a lack of evidence regarding its specific role in bridging the gap for pregnant surgeons to keep them in in training even in the period the surgical/sector room is forbidden by law, without keeping in training especially in what concerns manual dexterity. Italy's regulatory framework protects against gender disparities (40), supporting female workers and in particular surgeons during pregnancy by providing comprehensive maternity leave policies, job protection, and access to healthcare services. However, there remains a need for continuous improvement in workplace accommodations and the integration of technology to enhance safety and professional development for young female surgeons, including those in training as gynecologists or forensic pathologists. By ensuring that these professionals can continue practicing during pregnancy, while minimizing risks through innovative technologies, Italy can further advance gender health equity in surgical fields. Further studies are also needed to assess whether this is a real need for practicing pregnant surgeons.

## References

[1] Di Donna G Di Lorenzo P Aquino CI Marisei M Casella C Surico D Gender violence during the three ages of life and the impact of the COVID-19 pandemic: a review. Int J Soc Determinants Health Health Serv. (2024) 54(4):423–35. 10.1177/27551938241247776 38646684

[2] Tang JE Vassiliou MC Feldman LS. Fertility, family, and a career in surgery-time to change the narrative. JAMA Surg. (2024) 159(2):178. 10.1001/jamasurg.2023.6397 38091033

[3] Adesoye T Mangurian C Choo EK Girgis C Sabry-Elnaggar H Linos E Physician moms group study group. Perceived discrimination experienced by physician mothers and desired workplace changes: a cross-sectional survey. JAMA Intern Med. (2017) 177:1033–6. 10.1001/jamainternmed.2017.1394 28492824 PMC5818808

[4] Di Lorenzo P Casella C Marisei M Sarno L Aquino CI Osuna E A COVID dilemma: how to manage pregnancies in case of severe respiratory failure? Healthcare (Basel). (2023) 11(4):486. 10.3390/healthcare11040486 36833020 PMC9957288

[5] Simpson AN Cusimano MC Baxter NN. The inconvenience of motherhood during a medical career. CMAJ. (2021) 193(37):E1465–6. 10.1503/cmaj.211255 34544787 PMC8476216

[6] Cusimano MC Baxter NN Sutradhar R McArthur E Ray JG Garg AX Delay of pregnancy among physicians vs nonphysicians. JAMA Intern Med. (2021) 181(7):905–12. 10.1001/jamainternmed.2021.1635 33938909 PMC8094034

[7] Cusimano MC Baxter NN Sutradhar R Ray JG Garg AX McArthur E Reproductive patterns, pregnancy outcomes and parental leave practices of women physicians in Ontario, Canada: the dr mom cohort study protocol. BMJ Open. (2020) 10:e041281. 10.1136/bmjopen-2020-041281 33087379 PMC7580071

[8] Bering J Pflibsen L Eno C Radhakrishnan P. Deferred personal life decisions of women physicians. J Womens Health (Larchmt). (2018) 27:584. 10.1089/jwh.2016.6315 29634448

[9] Nasab S Shah JS Nurudeen K Jooya ND Abdallah ME Sibai BM. Physicians’ attitudes towards using elective oocyte cryopreservation to accommodate the demands of their career. J Assist Reprod Genet. (2019) 36:1935–47. 10.1007/s10815-019-01541-7 31376103 PMC6730980

[10] MUR. Health area graduate schools: here are the results of physicians’ evaluation (2021). Available online at: https://www.mur.gov.it/it/news/martedi-20072021/scuole-di-specializzazione-di-area-sanitaria-ecco-i-risultati-della (Accessed January 2, 2025).

[11] Gates M Gates A Pieper D Fernandes RM Tricco AC Moher D Reporting guideline for overviews of reviews of healthcare interventions: development of the PRIOR statement. Br Med J. (2022) 378:e070849. 10.1136/bmj-2022-070849 35944924 PMC9361065

[12] Stentz NC Griffith KA Perkins E Jones RD Jagsi R. Fertility and childbearing among American female physicians. J Womens Health (Larchmt). (2016) 25:1059–65. 10.1089/jwh.2015.5638 27347614

[13] Rangel EL Castillo-Angeles M Easter SR Atkinson RB Gosain A Hu YY Incidence of infertility and pregnancy complications in US female surgeons. JAMA Surg. (2021) 156(10):905–15. 10.1001/jamasurg.2021.3301. Erratum in: *JAMA Surg*. (2021) 156(10):991. doi: 10.1001/jamasurg.2021.4450 34319353 PMC9382914

[14] Giantini Larsen AM Pories S Parangi S Robertson FC. Barriers to pursuing a career in surgery: an institutional survey of harvard medical school students. Ann Surg. (2021) 273(6):1120–6. 10.1097/SLA.0000000000003618 31599803

[15] Finch SJ. Pregnancy during residency: a literature review. Acad Med. (2003) 78(4):418–28. 10.1097/00001888-200304000-00021 12691977

[16] Sayres M Wyshak G Denterlein G Apfel R Shore E Federman D. Pregnancy during residency. N Engl J Med. (1986) 314(7):418–23. 10.1056/NEJM198602133140705 3945268

[17] Rangel EL Smink DS Castillo-Angeles M Kwakye G Changala M Haider AH Pregnancy and motherhood during surgical training. JAMA Surg. (2018) 153(7):644–52. 10.1001/jamasurg.2018.0153 29562068 PMC5875346

[18] Hodson L Ovesen J Couch J Hirst D Lawson C Lentz TJ Managing Hazardous Drug Exposures: Information for Healthcare Settings. US Department of Health and Human Services, Centers for Disease Control and Prevention. National Institute for Occupational Safety and Health, DHHS (NIOSH) (2023). 10.26616/NIOSHPUB2023130

[19] Mann H Glazer T. Current state of safe pregnancy policies for the US surgical trainee. OTO Open. (2024) 8(3):e172. 10.1002/oto2.172 39036338 PMC11260283

[20] Hirayama M Fernando S. Organizational barriers to and facilitators for female surgeons’ career progression: a systematic review. J R Soc Med. (2018) 111(9):324–34. 10.1177/0141076818790661 30175935 PMC6146338

[21] Italian Regulation 151/2001. Single text of legislative provisions on the protection and support of maternity and paternity. Available online at: https://www.gazzettaufficiale.it/eli/gu/2001/04/26/96/so/93/sg/pdf (Accessed October 12 2024).

[22] Italian Regulation 81/2008. Implementation of Article 1 of Law No. 123 of August 3, 2007, on health and safety protection in the workplace. Available online at: https://www.gazzettaufficiale.it/eli/id/2008/04/30/008G0104/sg (Accessed on 12 October 2024).

[23] Gazzettaufficiale. State budget for the financial year 2020 and multi-year budget for the three-year period 2020–2022. Available online at: https://www.gazzettaufficiale.it/eli/id/2019/12/30/19G00165/s (Accessed October 12 2024).

[24] HamiltonLC. The lesser spotted pregnant surgeon. Ann R Coll Surg Engl. (2017). 10.1308/rcsann.2017.017729046076

[25] Europa Union. Council Directive 92/85/EEC of 19 October 1992 on the introduction of measures to encourage improvements in the safety and health at work of pregnant workers and workers who have recently given birth or are breastfeeding Current consolidated version: 26/07/2019. Available online at: https://eur-lex.europa.eu/legal-content/EN/TXT/?uri=celex%3A31992L0085 (Accessed October 17 2024).

[26] Europa Union. Directive 2006/54/EC of the European Parliament and of the Council of 5 July 2006 on the implementation of the principle of equal opportunities and equal treatment of men and women in matters of employment and occupation. Available online at: https://eur-lex.europa.eu/legal-content/EN/TXT/?uri=celex%3A32006L0054 (Accessed on 11 October 2024).

[27] Sterbling HM Kelly CH Stafford A Willey S Dort J. Pregnancy curriculum: advocating for a healthier pregnancy in general surgery residency. J Surg Educ. (2023) 80(12):1799–805. 10.1016/j.jsurg.2023.08.004 37661564

[28] Dowgiałło-Gornowicz N Zięty JJ Gornowicz M Sztaba K Osowiecka K Lech P. To be a pregnant surgeon—is there anything to be afraid of? Int J Environ Res Public Health. (2023) 20(3):2265. 10.3390/ijerph20032265 36767631 PMC9915432

[29] OECD. Paid parental leave: Big differences for mothers and fathers (2023). Available online at: https://www.oecd.org/en/blogs/2023/01/Paid-parental-leave–Big-differences-for-mothers-and-fathers.html (Accessed January 2, 2025).

[30] Jackson R Birsner ML Terman S Morris L. ACOG Committee opinion No. 733: employment considerations during pregnancy and the postpartum period. Obstet Gynecol. (2018) 131(4):e115–23. 10.1097/AOG.0000000000002589 29578986

[31] National Archives. Code of federal regulations the family and medical leave act (2013). Available online at: https://www.ecfr.gov/current/title-29/subtitle-B/chapter-V/subchapter-C/part-825/subpart-A/section-825.100 (Accessed January 2, 2025).

[32] National Archives. Federal register. Pregnant workers fairness act (2024). Available online at: https://www.federalregister.gov/documents/2024/04/19/2024-07527/implementation-of-the-pregnant-workers-fairness-act (Accessed January 2, 2025).

[33] EUR. Lex Council Directive 92/85/EEC of 19 October 1992 on the introduction of measures to encourage improvements in the safety and health at work of pregnant workers and workers who have recently given birth or are breastfeeding. Available online at: https://eur-lex.europa.eu/legal-content/EN/TXT/?uri=celex:31992L0085 (Accessed January 2, 2025).

[34] Mertens LS Pignot G Afferi L Vásquez JL Mir C Pradere B. Pregnancy and urology residency: towards equity-centred practice. Eur Urol. (2023) 84(2):152–3. 10.1016/j.eururo.2023.05.020 37268485

[35] Koven S Haberer JE Gomez Kwolek D. Pregnancy and residency—overdue for equity. N Engl J Med. (2023) 388(11):966–7. 10.1056/NEJMp2215288 36912537

[36] Marsters CM Stafl L Bugden S Gustainis R Nkunu V Reimer R Pregnancy, obstetrical and neonatal outcomes in women exposed to physician-related occupational hazards: a scoping review. BMJ Open. (2023) 13(2):e064483. 10.1136/bmjopen-2022-064483 36813500 PMC9950931

[37] Wang Y Li C Qu L Cai H Ge Y. Application and challenges of a metaverse in medicine. Front Robot AI. (2023) 10:1291199. 10.3389/frobt.2023.1291199 38152305 PMC10752600

[38] Wang Y Zhu M Chen X Liu R Ge J Song Y The application of metaverse in healthcare. Front Public Health. (2024) 12:1420367. 10.3389/fpubh.2024.1420367 39135928 PMC11317258

[39] Mazzoni D Pagin MM Amadori R Surico D Triberti S Aquino CI Chapter 6. The dual path of the technology acceptance model: an application of machine learning cardiotocography in delivery rooms. In: Artificial Intelligence for Medicine. (2024). 10.1016/B978-0-443-13671-9.00002-8

[40] Mazzoni D Pagin MM Amadori R Surico D Triberti S Aquino CI The dual path of the technology acceptance model: an application of machine learning cardiotocography in delivery rooms. In: Ben-David S Curigliano G Koff D Jereczek-Fossa BA La Torre D Pravettoni G, editors. An Applied Reference for Methods and Applications Advanced Studies in Complex Systems: Theory and Applications. Academic Press (2024). p. 73–80. 10.1016/B978-0-443-13671-9.00002-8

